# Supplementary material for: The relation between home numeracy practices and a variety of math skills in elementary school children
Source: PLoS One. 2021 Sep 20;16(9):e0255400. doi: 10.1371/journal.pone.0255400 (PMC8452026; doi:10.1371/journal.pone.0255400)
Supplement: S1 Appendix — (DOCX) [file pone.0255400.s001.docx]

**S1 Appendix. Subtests of the Zareki-R by math skill.**

**Quantity estimation**

- Numerosity estimation: Children have to approximate (i) the number of dots presented on two cards for 2 s each, (ii) the number of balls presented on one card for 5 s, and (iii) the number of glasses presented on one card for 5 s. The cards are presented too quickly for children to count.

- Quantity in context: Children must decide whether 10 scenarios (e.g., ‘‘four teachers in the same classroom,” ‘‘two clouds in the sky,” ‘‘10 leaves on a tree,”) depict something that is ‘‘a small number,” ‘‘an intermediate number,” or ‘‘a lot.”.

**Symbolic number understanding**

- Written comparison: Children are visually presented with 10 pairs of numbers and have to decide which is the largest. Pairs are: 13-31, 79-81, 1007-1070, 511-298, 654-546, 9768-35201, 96-69, 201-102, 347-947, and 1238-11238.

- Oral comparison: Children are orally presented with 10 pairs of numbers and have to decide which is the largest. Pairs are: 51-49, 465-546, 2009-2090, 108-800, 612-389, 800-108, 34601-9678, 46-64, and 1086-322.

- Number-to-position mapping: Children are presented with vertical number lines marked with 0 at the bottom and 100 at the top. They have to indicate where on the line a given number falls. Numbers are: 86, 48, 5, 32, 93, 62, 53, 27, 76, 17, 59, and 83.

**Counting**

- Dot counting: Children count the number of dots presented on six cards (with 13, 5, 15, 10, 8, and 18 dots).

- Oral reverse counting: Children count backwards from a given number to a stop number (e.g., from 67 to 54) and from a given number without stop number (e.g., from 23 to 1).

**Transcoding**

- Number reading: Children have to read eight Arabic numbers (i.e., 15, 57, 1900, 305, 138, 6485, 687, and 969).

- Number writing: Children have to write eight numbers spoken by the experimenter (i.e., 14, 38, 1200, 503, 169, 4658, 756, and 689).

**Arithmetic calculation**

- Oral problem solving: Children are orally presented with six number problems that they have to solve (e.g., ‘‘Peter has 12 marbles. He gives 5 to his friend Ann. How many marbles does Peter have now?”). Problems increase in difficulty.

- Addition: Children have to solve eight addition problems presented orally (i.e., 5 + 8, 12 + 6, 4 + 13, 9 + 7, 15 + 12, 13 + 19, 14 + 8, and 17 + 25).

- Subtraction: Children have to solve eight subtraction problems presented orally (i.e., 17 - 5, 14 - 6, 24 - 17, 19 - 6, 15 - 9, 25 - 12, 32 - 17, and 18 - 11).

- Multiplication: Children have to solve six multiplication problems presented orally (i.e., 3 × 2, 4 × 5, 3 × 4, 2 × 6, 5 × 3, and 4 × 4).
