# Supplementary material for: The relation between home numeracy practices and a variety of math skills in elementary school children
Source: PLoS One. 2021 Sep 20;16(9):e0255400. doi: 10.1371/journal.pone.0255400 (PMC8452026; doi:10.1371/journal.pone.0255400)
Supplement: S1 Table — (DOCX) [file pone.0255400.s002.docx]

**S1 Table. Formal numeracy activities and approximate grades in which they are mainly practiced in school according to the French math curriculum.**

| Skill level | Item | Grade |
| --- | --- | --- |
|  |  |  |
| Basic | Counting objects | Preschool - Kindergarten |
|  | Counting without objects | Kindergarten - Grade 1 |
|  | Memorizing results of simple addition problems | Grade 1 - 2 |
|  | Memorizing multiplication tables | Grade 1 - 2 - 3 |
|  | Comparing quantities | Kindergarten - Grade 1 |
|  | Adding numbers | Grade 1 - 2 |
|  | Subtracting single-digit numbers (e.g., 8-1) | Grade 1 - 2 |
|  | Multiplying single-digit numbers (e.g., 2x3) | Grade 1 - 2 |
|  | Talking about sharing | Grade 1 - 2 - 3 |
|  | Dividing small numbers (e.g., 6÷2) | Grade 1 - 2 - 3 |
|  | Writing numbers up to 20 | Kindergarten - Grade 1 |
|  | Writing numbers up to 100 | Grade 1 - 2 |
|  | Reading numbers up to 20 | Kindergarten - Grade 1 |
|  | Reading numbers up to 100 | Grade 1 - 2 |
|  |  |  |
| Advanced | Subtracting double-digit numbers (e.g., 34-16) | Grade 2 - 3 - 4 |
|  | Multiplying double-digit numbers (e.g., 12x6) | Grade 3 - 4 |
|  | Dividing double-digit numbers (e.g., 12÷4) | Grade 3 - 4 - 5 |
|  | Writing numbers up to 1,000 | Grade 2 - 3 |
|  | Reading numbers up to 1,000 | Grade 2 - 3 |
|  |  |  |
