# Supplementary material for: The relation between home numeracy practices and a variety of math skills in elementary school children
Source: PLoS One. 2021 Sep 20;16(9):e0255400. doi: 10.1371/journal.pone.0255400 (PMC8452026; doi:10.1371/journal.pone.0255400)
Supplement: S2 Table — (DOCX) [file pone.0255400.s003.docx]

**S2 Table. Frequency ratings associated with informal home literacy practices.**

| **Item** | **Mean (SD)** | **Min** | **Max** |
| --- | --- | --- | --- |
|  |  |  |  |
| **Telling invented stories^2^** | 1.15 (1.22) | 0 | 5 |
| **Talking about school day^2^** | 4.64 (0.57) | 3 | 5 |
| **Playing computer/tablet games involving reading or spelling^1^** | 0.65 (0.83) | 0 | 4 |
| **Reading texts in everyday life (advertisement, etc.) ^2^** | 3.71 (1.31) | 0 | 5 |
| **Visiting the library for children’s books^1^** | 1.24 (0.8) | 0 | 5 |
| **Singing songs^2^** | 2.91 (1.61) | 0 | 5 |
| **Singing alphabet songs^2^** | 0.89 (0.78) | 0 | 5 |
| **Making up rhymes^2^** | 1.39 (1.2) | 0 | 5 |
| ***Average*** | *2.07 (1.76)* |  |  |
|  |  |  |  |

Minimum rating is 0, maximum rating is 5. All parents (N=66) were presented with all of the items.

^1^Items directly translated from the LeFevre et al.’s questionnaire.

^2^Items adapted from the LeFevre et al.’s questionnaire to account for the fact that children in the present study are older.
