# Supplementary material for: The relation between home numeracy practices and a variety of math skills in elementary school children
Source: PLoS One. 2021 Sep 20;16(9):e0255400. doi: 10.1371/journal.pone.0255400 (PMC8452026; doi:10.1371/journal.pone.0255400)
Supplement: S3 Table — (DOCX) [file pone.0255400.s004.docx]

**S3 Table. Frequency ratings associated with formal home literacy practices.**

| **Skill level** | **Item** | **Mean (SD)** | **Min** | **Max** | **N** |
| --- | --- | --- | --- | --- | --- |
|  |  |  |  |  |  |
| **Basic** | **Writing short texts^2^** | 0.85 (0.8) | 0 | 4 | 66 |
|  | **Reading short texts^2^** | 3.03 (1.62) | 1 | 5 | 66 |
|  | **Listening while my child reads books out loud^1^** | 2.55 (1.51) | 0 | 5 | 66 |
|  | **Reading together^1^** | 2.14 (1.37) | 0 | 5 | 66 |
|  | ***Average*** | *2.14 (1.59)* |  |  | 66 |
|  |  |  |  |  |  |
| **Advanced** | **Writing long texts^2^** | 0.36 (0.54) | 0 | 2 | 44 |
|  | **Reading long texts^2^** | 2.2 (1.46) | 0 | 5 | 66 |
|  | **Asking questions when my child reads alone ^2^** | 2.35 (1.43) | 0 | 5 | 66 |
|  | **Asking questions when we read together^1^** | 1.8 (1.35) | 0 | 5 | 66 |
|  | **Teaching and correcting spelling^2^** | 2.98 (1.38) | 0 | 5 | 66 |
|  | **Teaching and correcting conjugation^2^** | 2.33 (1.28) | 0 | 5 | 66 |
|  | ***Average*** | 2.01 (*1.52)* |  |  |  |
|  | | | | | |

N, number of parents who were presented with the item; Minimum rating is 0, maximum rating is 5. ^1^Items directly translated from the LeFevre et al.’s questionnaire.

^2^Items adapted from the LeFevre et al.’s questionnaire to account for the fact that children in the present study are older.
