# Supplementary material for: The relation between home numeracy practices and a variety of math skills in elementary school children
Source: PLoS One. 2021 Sep 20;16(9):e0255400. doi: 10.1371/journal.pone.0255400 (PMC8452026; doi:10.1371/journal.pone.0255400)
Supplement: S4 Table — (DOCX) [file pone.0255400.s005.docx]

**S4 Table. Effect sizes and t-values associated with multiple regression analyses of numeracy practices on math subtests across the subset of participants who were all presented with the frequency items “subtracting double-digit numbers” and “multiplying double-digit numbers”.**

| **Practice** | **Quantity Estimation^1^** | | **Symbolic number understanding^1^** | | **Counting^1^** | | **Transcoding^1^** | | **Arithmetic calculation^1^** | | **Arithmetic fluency^2^** | |
| --- | --- | --- | --- | --- | --- | --- | --- | --- | --- | --- | --- | --- |
|  | **η²p** | **t** | **η²p** | **t** | **η²p** | **t** | **η²p** | **t** | **η²p** | **t** | **η²p** | **t** |
| **Informal** | 0.004 | -0.481 | 0.010 | 0.747 | 0.020 | 1.049 | 0.012 | 0.814 | 0.025 | 1.169 | 0.039 | -1.482 |
| **Formal basic** | 0.001 | 0.194 | 0.004 | -0.459 | 0.068 | -1.977 | 0.004 | -0.463 | **0.098** | **-2.428** | 0.022 | -1.103 |
| **Formal advanced** | 0.008 | 0.663 | 0.001 | 0.249 | 0.004 | 0.469 | 0.013 | -0.849 | **0.155** | **3.144** | **0.112** | **2.603** |
|  |  |  |  |  |  |  |  |  |  |  |  |  |
| **R^2^** | 0.016 | | 0.013 | | 0.076 | | 0.035 | | 0.188 | | 0.131 | |

N=58; p < .05 (two-tailed) in bold; η2ps represent effect sizes that can be considered small (0.01), medium (0.06), or large (0.14).

^1^Zareki-R.

^2^WJ-III.
