# Supplementary material for: The relation between home numeracy practices and a variety of math skills in elementary school children
Source: PLoS One. 2021 Sep 20;16(9):e0255400. doi: 10.1371/journal.pone.0255400 (PMC8452026; doi:10.1371/journal.pone.0255400)
Supplement: S6 Table — (DOCX) [file pone.0255400.s007.docx]

| Practice | Quantity Estimation^1^ | | Symbolic number understanding^1^ | | Counting^1^ | | Transcoding^1^ | | Arithmetic calculation^1^ | | Arithmetic fluency^2^ | |
| --- | --- | --- | --- | --- | --- | --- | --- | --- | --- | --- | --- | --- |
|  | η²p | t | η²p | t | η²p | t | η²p | t | η²p | t | η²p | t |
|  | | | | | | | | | | | | |
| *Present numeracy practices with parent* | | | | | | | | | | | | |
|  | | | | | | | | | | | | |
| Informal | 0.002 | -0.37 | 0.013 | 0.863 | 0.021 | 1.106 | 0.026 | 1.208 | 0.041 | 1.548 | 0.002 | -0.305 |
| Formal basic | 0.007 | 0.64 | <0.001 | -0.073 | 0.025 | -1.196 | <0.001 | -0.059 | 0.053 | -1.779 | 0.011 | -0.772 |
| Formal advanced | 0.002 | 0.35 | 0.015 | 0.920 | 0.002 | 0.305 | 0.002 | -0.346 | 0.061 | 1.908 | **0.093** | **2.399** |
|  |  |  |  |  |  |  |  |  |  |  |  |  |
| *Present numeracy practices without parent* | | | | | | | | | | | | |
|  | | | | | | | | | | | | |
| Informal | 0.004 | -0.50 | 0.038 | -1.487 | 0.001 | 0.268 | 0.017 | 0.977 | 0.005 | 0.519 | <0.001 | -0.016 |
| Formal basic | 0.018 | -1.01 | <0.001 | 0.137 | 0.015 | -0.915 | 0.003 | 0.407 | 0.012 | 0.834 | 0.033 | -1.375 |
| Formal advanced | **0.081** | **2.22** | 0.043 | 1.586 | 0.030 | 1.306 | 0.006 | 0.577 | 0.006 | 0.595 | **0.118** | **2.741** |
|  |  |  |  |  |  |  |  |  |  |  |  |  |
| *Past numeracy practices with parent* | | | | | | | | | | | | |
|  | | | | | | | | | | | | |
| Informal | 0.042 | 1.57 | <0.001 | -0.099 | 0.001 | -0.181 | <0.001 | 0.129 | 0.002 | -0.336 | 0.003 | -0.381 |
| Formal basic | 0.031 | -1.34 | 0.004 | 0.453 | 0.015 | -0.922 | 0.005 | 0.524 | 0.005 | -0.538 | 0.008 | -0.654 |
| Formal advanced | 0.026 | 1.22 | **0.080** | **2.201** | **0.071** | **2.068** | 0.017 | 0.992 | 0.029 | 1.297 | **0.093** | **2.390** |
|  |  |  |  |  |  |  |  |  |  |  |  |  |
| R^2^ | 0.148 | | 0.195 | | 0.120 | | 0.133 | | 0.198 | | 0.226 | |

**S6 Table. Effect sizes and t-values associated with multiple regression analyses of present and past numeracy practices on math subtests across all participants.**

N=66; p < .05 (two-tailed) in bold. η2ps represent effect sizes that can be considered small (0.01), medium (0.06) or large (0.14) (83).

^1^, Zareki-R.

^2^, WJ-III.
