# Supplementary material for: The relation between home numeracy practices and a variety of math skills in elementary school children
Source: PLoS One. 2021 Sep 20;16(9):e0255400. doi: 10.1371/journal.pone.0255400 (PMC8452026; doi:10.1371/journal.pone.0255400)
Supplement: S7 Table — (DOCX) [file pone.0255400.s008.docx]

| **Literacy practice** | **Quantity Estimation^1^** | | **Symbolic number understanding^1^** | | **Counting^1^** | | **Transcoding^1^** | | **Arithmetic calculation^1^** | | **Arithmetic fluency^2^** | |
| --- | --- | --- | --- | --- | --- | --- | --- | --- | --- | --- | --- | --- |
|  | **η²p** | **t** | **η²p** | **t** | **η²p** | **t** | **η²p** | **t** | **η²p** | **t** | **η²p** | **t** |
| **Informal** | 0.007 | 0.684 | 0.015 | -0.971 | 0.006 | 0.591 | 0.026 | 1.275 | 0.010 | -0.795 | 0.039 | -1.595 |
| **Formal basic** | <.001 | -0.152 | <.001 | -0.085 | 0.002 | 0.334 | <.001 | 0.078 | 0.004 | -0.512 | 0.008 | -0.704 |
| **Formal advanced** | 0.008 | -0.717 | 0.008 | -0.722 | 0.023 | -1.198 | 0.028 | -1.329 | 0.008 | 0.702 | 0.025 | 1.268 |
|  |  |  |  |  |  |  |  |  |  |  |  |  |
| **R^2^** | 0.018 | | 0.043 | | 0.027 | | 0.050 | | 0.019 | | 0.063 | |

**S7 Table. Effect sizes and t-values associated with multiple regression analyses of literacy practices on math subtests across all participants.**

N=66; p < .05 (two-tailed) in bold; η2ps represent effect sizes that can be considered small (0.01), medium (0.06), or large (0.14).

^1^Zareki-R.

^2^WJ-III.
