# Supplementary material for: The relation between home numeracy practices and a variety of math skills in elementary school children
Source: PLoS One. 2021 Sep 20;16(9):e0255400. doi: 10.1371/journal.pone.0255400 (PMC8452026; doi:10.1371/journal.pone.0255400)
Supplement: S8 Table — (DOCX) [file pone.0255400.s009.docx]

**S8 Table. Pearson correlation coefficients between present and past home numeracy practices and parental traits across all participants.**

|  | **1.** | **2.** | **3.** | **4.** | **5.** | **6.** | **7.** | **8.** | **9.** | **10.** | **12.** | **13.** | **14.** | **15.** | **16.** | **17.** |
| --- | --- | --- | --- | --- | --- | --- | --- | --- | --- | --- | --- | --- | --- | --- | --- | --- |
| ***Present numeracy practices with parent*** |  |  |  |  |  |  |  |  |  |  |  |  |  |  |  |  |
| **1. Informal** |  |  |  |  |  |  |  |  |  |  |  |  |  |  |  |  |
| **2. Formal / basic** | 0.49*** |  |  |  |  |  |  |  |  |  |  |  |  |  |  |  |
| **3. Formal / advanced** | 0.30** | 0.76*** |  |  |  |  |  |  |  |  |  |  |  |  |  |  |
|  |  |  |  |  |  |  |  |  |  |  |  |  |  |  |  |  |
| ***Present numeracy practices without parent*** |  |  |  |  |  |  |  |  |  |  |  |  |  |  |  |  |
| **4. Informal** | -0.16 | 0.10 | 0.11 |  |  |  |  |  |  |  |  |  |  |  |  |  |
| **5. Formal / basic** | 0.05 | -0.04 | 0.03 | 0.31** |  |  |  |  |  |  |  |  |  |  |  |  |
| **6. Formal / advanced** | -0.03 | -0.13 | -0.05 | 0.22* | 0.77*** |  |  |  |  |  |  |  |  |  |  |  |
|  |  |  |  |  |  |  |  |  |  |  |  |  |  |  |  |  |
| ***Past numeracy practices with parent*** |  |  |  |  |  |  |  |  |  |  |  |  |  |  |  |  |
| **7. Informal** | -0.26 | -0.26 | -0.19 | 0.04 | -0.03 | -0.10 |  |  |  |  |  |  |  |  |  |  |
| **8. Formal / basic** | -0.35 | -0.42 | -0.28 | 0.03 | -0.35 | -0.23 | 0.45*** |  |  |  |  |  |  |  |  |  |
| **9. Formal / advanced** | -0.20 | -0.27 | -0.37 | 0.09 | -0.24 | -0.29 | 0.30** | 0.65*** |  |  |  |  |  |  |  |  |
|  |  |  |  |  |  |  |  |  |  |  |  |  |  |  |  |  |
| ***Parental traits*** |  |  |  |  |  |  |  |  |  |  |  |  |  |  |  |  |
| **10. Parental education** | -0.14 | -0.31 | -0.12 | 0.12 | 0.14 | 0.13 | 0.08 | 0.28* | 0.14 |  |  |  |  |  |  |  |
| **11. Parental income** | 0.03 | -0.12 | -0.11 | 0.04 | -0.14 | -0.12 | -0.08 | 0.28** | 0.14 | 0.41*** |  |  |  |  |  |  |
| **12. Parental basic expectations** | 0.13 | -0.01 | 0.09 | <0.01 | -0.06 | -0.06 | 0.17† | 0.25* | 0.16 | 0.09 | 0.11 |  |  |  |  |  |
| **13. Parental advanced expectations** | 0.21* | 0.26* | 0.33** | 0.02 | -0.13 | -0.10 | 0.02 | 0.13 | 0.06 | -0.10 | 0.03 | 0.56*** |  |  |  |  |
| **14. Parental math attitude** | -0.11 | -0.13 | -0.03 | 0.03 | -0.11 | -0.15 | 0.10 | 0.24* | 0.18† | 0.45*** | 0.24* | 0.12 | 0.12 |  |  |  |
| **15. Parental arithmetic fluency** | 0.02 | -0.03 | -0.10 | <0.01 | -0.05 | -0.01 | <0.01 | 0.18† | 0.13 | 0.56*** | 0.37** | 0.17† | -0.03 | 0.42*** |  |  |
| **16. Numbers of hours spent with the child** | 0.03 | 0.11 | 0.08 | 0.05 | -0.03 | -0.12 | 0.09 | 0.05 | 0.03 | -0.03 | -0.36 | 0.27* | 0.10 | -0.01 | -0.03 |  |
| **17. Parental estimates of the child’s math skills** | 0.06 | 0.01 | 0.02 | 0.10 | 0.02 | 0.05 | <0.01 | 0.04 | -0.08 | 0.07 | 0.09 | 0.22* | 0.12 | 0.21* | 0.09 | 0.08 |

N=66; ***, p < .001; **, p < .01; *, p < .05., †, p < .1. P values are one-tailed (testing for a positive association).
