# Supplementary material for: The relation between home numeracy practices and a variety of math skills in elementary school children
Source: PLoS One. 2021 Sep 20;16(9):e0255400. doi: 10.1371/journal.pone.0255400 (PMC8452026; doi:10.1371/journal.pone.0255400)
Supplement: S9 Table — (DOCX) [file pone.0255400.s010.docx]

| Predictor | Informal practices | | Formal basic practices | | Formal advanced practices | |
| --- | --- | --- | --- | --- | --- | --- |
|  | η²p | t | η²p | t | η²p | t |
| Parental arithmetic fluency | 0.007 | 0.654 | 0.010 | 0.774 | 0.002 | -0.384 |
| Parental basic expectations | <.001 | 0.082 | 0.041 | -1.618 | 0.011 | -0.807 |
| Parental advanced expectations | 0.036 | 1.500 | **0.115** | **2.822** | **0.109** | **2.733** |
| Parental attitudes | 0.026 | -1.267 | 0.034 | -1.463 | 0.002 | -0.321 |
|  |  |  |  |  |  |  |
| R^2^ | 0.070 | | 0.131 | | 0.129 | |

**S9 Table. Standardized coefficients, 95% confidence intervals (CI), and effect sizes for the multiple regression analysis of present numeracy practices.**

N=66; p < .01 (two-tailed) in bold; η2ps represent effect sizes that can be considered small (0.01), medium (0.06) or large (0.14) (83).
