# Supplementary material for: The relation between home numeracy practices and a variety of math skills in elementary school children
Source: PLoS One. 2021 Sep 20;16(9):e0255400. doi: 10.1371/journal.pone.0255400 (PMC8452026; doi:10.1371/journal.pone.0255400)
Supplement: Attachment — Submitted filename: Reponse_to_Reviewers.doc [file pone.0255400.s013.doc]

Responses to reviews

We wish to thank Dr. van den Boer and the reviewers for their thoughtful and helpful suggestions. Below, we indicate the specific responses to the issues raised and the corresponding changes that we have made in the revised manuscript.

**Dr. van den Boer**

*Both reviewers are very positive about the manuscript, but suggest (rather small) changes to improve the overall quality of the paper. As the reviewers have been very clear in their suggestions for improvement I will not repeat them here. In addition to the suggestions of the reviewers, there are three remaining issues from my own reading of the manuscript that hopefully could be addressed in a revision:*

*- Using the abbreviation HNE in the Abstract was a bit confusing to me, as it only becomes clear in the main text what it stands for. Could HNE be written in full in the Abstract?*

Response: This has now been corrected.

*- I would very much prefer it if the correlations could be provided in the main text, as these form the basis of the analyses.*

Response: We have now added in the main text the Pearson correlation matrices that are at the basis of our analyses (see Table 5 for the correlations between home numeracy practices and math scores and Table 6 for the correlations between home numeracy practices and parental traits).

*- Little information is provided about the analyses. Some elaboration on the chosen strategy of analysis would be very helpful.*

Response: We thank the editor for this comment. We have now added a “Data analysis” section in the Method (p. 19). There we state:

*“*The relations between all math skills and the 3 types of home numeracy practices (informal, formal basic and formal advanced) were first explored using bivariate Pearson correlations and subsequently tested using multiple regression analyses with frequency of informal, formal basic and formal advanced practices as predictors and scores on math subtests as outcomes. To test whether any of the relations observed were explained by other measured variables, we then adjusted these regressions for children’s overall cognitive functioning, parental SES, parental math skills, and time spent with the child at home.

Finally, the relations between home numeracy practices and parental traits were also explored using Pearson correlations and subsequently tested using multiple regression analyses with parental traits (parental arithmetic fluency, basic and advanced expectations towards math and math attitudes) as predictors and reported frequency of the home numeracy practices as outcomes. These regressions were also adjusted for parental SES, parental estimates of their child’s math skills time spent with the child at home. All analyses were conducted in Jamovi version 1.6.3.”

**Reviewer 1**

*(1) The literature review is well-grounded, however, the way in which the literature ties together is unclear. Beginning each paragraph with “second”, “third”, “fourth” does not inherently tie the pieces together. It would be more appropriate to use transition statements across the broad headings in the introduction and clarify why each subheading builds upon the next (if it does).*

Response: We are grateful to the reviewer for this comment, which helped us improve the introduction. We have now revised our introduction to make better use of transition statements and clarify the structure (see p. 4 to p. 8).

*(2) Currently, your headings seem more like place holders rather than formal subheadings. For example can “Relation between the HNE and Math skills in elementary school” become “HNE and Math Skills in Elementary School”?*

Response: We have followed the reviewer’s advice and have changed our headings in the introduction so that they are more formal (see p. 4 to p. 7).

*(3) Line 74-76 states studies have found the relation or have failed, but what about studies that have found a significant negative relation (Skwarchuk et al., 2014)?*

Response: We thank the reviewer for pointing this out. We now explicitly mention these studies on p. 4. There, we state:

“However, the relation between math skills and the HNE appears to be relatively complex and sometimes inconsistent (for a review, see (25,26). For example, several studies have found a relation between higher quality HNE (i.e., more frequent home numeracy activities) and better numerical skills (e.g., 8,10,19,20,22,24,27–32). However, other studies have failed to find such an association (e.g., 33–36) and some have even found a negative relation (22,37). How can this relative inconsistency be explained?”

*(4) Hypotheses are not stated in the manuscript. It would be helpful if the dense literature review in the introduction was used to inform your hypotheses.*

Response: Our hypotheses are now more clearly stated at the end of the introduction. On p 8-9, we now state:

“This extensive data collection allowed us to address two main questions. First, we asked whether there remains a relation between home numeracy practices and math skills in these children, who are older than children typically tested in the HNE literature. Critically, our range of measures allowed us to (a) control for parental math skills, (b) assess which numeracy practices (informal versus formal, basic versus advanced) relate to specific math skills, and (c) evaluate whether the relation would be specific to numeracy practices (i.e., not observed with comparable literacy practices). In keeping with the previous literature on younger children, we expected to observe a relation between the HNE and math skills that would depend upon the type and difficulty of the practice. That is, we anticipated that informal practices would be associated with non-symbolic skills while formal practices would be associated with symbolic skills, especially when those were relatively advanced with respect to children’s age.

Second, we also aimed to investigate which parental traits (e.g., expectations, attitudes, skills) predict more frequent numeracy practices. Indeed, several studies have investigated the role of parental math attitude and academic expectations in shaping the HNE (see 25,26). Parental attitudes include their level of math anxiety (i.e., their tendency to avoid situations that involve math, 70) but also their use of math in everyday life as well as their evaluation of their own math skills when they were in school (71,72). Academic expectations are typically measured by asking parents how important it is for them that their child acquires a given skill within some time frame (e.g., 22). Overall, although an association between parental attitudes and home numeracy practices remains to be clearly demonstrated (e.g., 20,22), studies have more consistently found a relation between higher academic expectations and increased frequency of numeracy practices at home (18,19,22,30,43). Thus, we expected that the frequency of math activities reported by parents would depend on their academic expectations.” *(5) The exclusion criteria make sense for neuroimaging methods, but why were these same exclusion criteria included for behavioral, home environment analyses? Make this clearer why you think these exclusions matter for your research questions.*

Response: We thank the reviewer for this comment and have now specified why we used these exclusion criteria. On p. 9, we state:

“Because studies suggest that the home learning environment may differ in children with low intelligence quotient (IQ) (73) and attention deficit disorder (74), we excluded children who had an IQ lower than the 25th percentile (n=2) and were diagnosed with attention deficit disorder (n=1). Because we also anticipated differences in learning environment of children who experienced and were followed for language difficulties (75), we also excluded children who were seeing a speech-language pathologist on a regular basis (n=3) and were diagnosed with attention deficit disorder (n=1).”

*(6) For participant age, just report mean/min/max rather than single out participants*

Response: We have followed the reviewer’s advice. On p. 10, we state:

*“*Therefore, 66 typically-developing 8-year-olds (M=8.46, min=7.51, max=9.22) with no history of neurological disease, mental disorders of attention deficits were included in the final analyses.” *(7) I appreciate your power analysis! Can you cite which tool or calculation you used?*

Response: We thank the reviewer for this comment. We used G*Power (Faul et al., 2007). We added the reference in the manuscript and some more detail about the calculation. On p. 10, we state:

“Considering the smallest estimate of that range (r=0.31), G*Power 3.1 (76) indicated that our final sample (n=66) would provide an achieved power of 84% to detect a similar positive association in a linear regression at α = 0.05 (one tailed).” *(8) Like the authors, I also felt a little uneasy about assuming parents did not do advanced math if they didn’t do basic. However, the authors have noted this as a limitation, and conducted robustness checks to ensure this design did not change findings presented in this manuscript. I really appreciate your thoughtfulness in this and your willingness to provide these robustness checks on OSF. Thank you!!*

Response: We thank the reviewer for the comment.

*(9) Line 603-605: a paragraph consists of at least 3 sentences. Please consider elaborating on this point in the limitations.*

Response: We thank the reviewer for the suggestion. We have now extended this paragraph. It now reads (on p. 29):

“A second limitation is that, although all children were extensively tested on a broader range of measures that what is typically found in the HNE literature, the overall sample size remains limited. Therefore, our results will need to be replicated in larger samples. Nonetheless, given the relative scarcity of data about effects of the HNE on children’s math skills outside of the US, we also believe that our data are valuable and may inform future meta-analysis.” *(10) The points of limitation are important, however, I also noticed that there was no discussion regarding home practices beyond numeracy (shapes/pattern; see Zippert & Rittle-Johnson, 2020 for review). This may be important to address in the limitations, as not much is known about these topics in older children.*

Response: This is good point and we thank the reviewer for bringing it up. We have now added this as a limitation. On p. 30 we state:

“A fourth limitation is that our questionnaire focused on activities that involved numerical content per se. However, math learning has been found to be associated with the development of other non-numerical skills, such as patterning and spatial processing (94,106). For example, it has been shown that children’s exposure to spatial activities at home is related to arithmetic skills in first grade (107). Therefore, future studies should assess the effect of home practices beyond numeracy on math skills of older children.”

*(11) In conclusions, “overall, our study adds to the literature on the HNE by showing that the relation between home numeracy practices and math skills is not restricted to the environment of young children” seems as though someone said that it did. I think what you mean here is rather the HNE for older children is also important for math skills, however, that is not what comes across in this last paragraph. Further, the next sentence is a run-on sentence and does not give a strong takeaway message from the interesting results of your study! Please work on re-wording and emphasizing your main results.*

Response: We have followed the reviewer’s advice and have reworded the conclusion. We also end on a sentence that emphasizes an important implication of our study. On p. 31-32, this revised section reads:

“Overall, our study adds to the literature on the HNE by showing that the relation between home numeracy practices and math skills remains present in elementary school children. Although our findings are correlational in nature, they broadly support interventions that may attempt to raise math skills of elementary school children by involving parents and caregivers.”

*- Line 644 asenxiety should be anxiety*

Response: This has been corrected

*- Line 278 missing the word measure*

Response: This has been corrected.

*- In Table 2, include the likert scale meanings in the Notes, same for 3 and 4*

Response: Likert scale meanings have been added in Tables 2, 3 and 4.

**Reviewer 2**

*(1) Could the authors include a table with the bivariate correlations between all their variables?*

Response: We have now added in the main text the Pearson correlation matrices that are at the basis of our analyses (see Table 5 for the correlations between home numeracy practices and math scores and Table 6 for the correlations between home numeracy practices and parental traits).

*(2) I think it would be helpful to know how what each questionnaire response option was numerically coded.*

Response: We have now added Likert scale meanings in Tables 2, 3 and 4. Additionally, we now provide more details in the main text. On p. 13, we state for the home numeracy questionnaire:

“Following LeFevre et al. (20), a six-point rating scale was used: Did not occur/Activity is not relevant to my child (coded 0), 1-3 times per month (coded 1), Once per week (coded 2), 2-4 times per week (coded 3), Almost daily (coded 4), Daily (coded 5). Given that children in the present study were older than children in LeFevre et al. (20), we added two other response options. First, if parents used to engage in a given activity in the past but no longer did at the time of testing, they could say so. Second, if parents had never engaged in the activity with the child but the child was doing that activity on her own, they could also say so. Each of these options was coded with the lowest non-0 rating (i.e., 1), as they might indicate a somewhat supportive home numeracy environment but do not convey any specific information about frequency (they therefore have minimal weigh on the overall final rating).”

On p. 17, we also state for the academic expectation questionnaire:

“First, because our questionnaire was presented in an electronic format, we added a “no opinion” option to the original scale, such that it had 6 options: Really not important (coded -3), Not important (coded -1), No opinion (coded 0), Important (coded 1), Very important (coded 2), Extremely important (coded 3).”

*(3) Can the authors give some explanation for why they coded engaging in an activity before but not now, and never engaged in the activity but the child did as a score of 1? This was something I was left concerned about, especially if 1 was also coded for a different response option. Are those options the same as “did not occur”? I’m not sure!*

Response: We thank the reviewer for this comment. These options are coded as 1 and not as “did not occur” (which is coded as 0) as they still indicate a somewhat supportive home environment. We now explain more clearly the rationale for this decision on p. 13. There, we state:

“Given that children in the present study were older than children in LeFevre et al. (20), we added two other response options. First, if parents used to engage in a given activity in the past but no longer did at the time of testing, they could say so. Second, if parents had never engaged in the activity with the child but the child was doing that activity on her own, they could also say so. Each of these options was coded with the lowest non-0 rating (i.e., 1), as they might indicate a somewhat supportive home numeracy environment but do not convey any specific information about frequency (they therefore have minimal weigh on the overall final rating).”
