# Supplementary material for: The relation between home numeracy practices and a variety of math skills in elementary school children
Source: PLoS One. 2021 Sep 20;16(9):e0255400. doi: 10.1371/journal.pone.0255400 (PMC8452026; doi:10.1371/journal.pone.0255400)
Supplement: Attachment — Submitted filename: Reponse_to_Reviewers.doc [file pone.0255400.s014.doc]

Responses to reviews

We wish to thank Dr. van den Boer and reviewer #2 for their further comments on the manuscript. Below, we indicate the specific responses to the issues raised and the corresponding changes that we have made in the revised manuscript.

**Dr. van den Boer**

*on page 9 (lines 199 and 202) a participant with ‘attention deficit disorder’ seems to be mentioned twice*

Response: We thank the editor for spotting this inconsistency. This was indeed an error. Only one participant was excluded for attention deficit disorder. This has been corrected in the new version of the manuscript. On p. 8/9, this section now reads:

“Because studies suggest that the home learning environment may differ in children with low intelligence quotient (IQ) (73) and attention deficit disorder (74), we excluded children who had an IQ lower than the 25th percentile (n=2) and were diagnosed with attention deficit disorder (n=1). Because we also anticipated differences in the learning environment of children who experienced and were followed for language difficulties (75), we also excluded children who were seeing a speech-language pathologist on a regular basis (n=3) and had a delay in speech and language acquisition (n=1).”

*on page 17 (lines 344/345) it is not clear to me how the added answer options are related to the fact that the questionnaire was administered in an electronic format… maybe the sentence could be reworded?*

Response: What we meant here is that because the questionnaire was in an electronic format, participants were forced to provide an answer to each item. Adding a ‘no opinion’ option gave them the opportunity to skip items if they wished to do so. This has now been clarified in the manuscript. On p. 16, we state:

“First, because our questionnaire was presented in an electronic format, parents were forced to give an answer to each item. Therefore, we added a “no opinion” option to the original scale to give them the option to skip an item if they wished to do so. In other words, there were 6 options for each item: Really not important (coded -3), Not important (coded -1), No opinion (coded 0), Important (coded 1), Very important (coded 2), Extremely important (coded 3).”

**Reviewer 2**

*Now that I know how the variables were coded, and see the rationale for the recoding of the HNE frequency questionnaire, I am more skeptical about the idea of the extra two responses options for the HNE (engaging in an activity before but not now, and never engaged in the activity but the child did) being coded as 1, the same as a low frequency option. To me this doesn’t seem to be a valid choice given the scale is a frequency scale, and also this choice means this work cannot be compared to other HNE work. I personally think these options should not be used at all, but given the authors likely disagree, I believe that at the very least they should do a sensitivity analysis to see if it matters. I would like to know if they recoded those response to missing, what their correlation and regression results would look like (and I think they should be provided somewhere for future meta-analysists).*

Response: We acknowledge that these two responses are typically not added to measures of the HNE in other studies. This is most certainly because the large majority of previous studies to date have focused on younger children who are less likely to either engage in numeracy activities by themselves or for whom most activities remain relevant. Given that children in our sample are 8 years old, it was critical for us to ask parents why they did not state that an activity was practiced during the past month. This choice was notably motivated by Lefevre et al. (LeFevre et al., 2010) who, when discussing discrepancy with an earlier study involving school-age children (LeFevre et al., 2009), observed that “basic skills were probably already mastered by many of the children in LeFevre et al. (who were in Kindergarten, Grade 1 and Grade 2) and thus were no longer relevant to predicting their more advanced numeracy skills.” Similarly, school-age children are more likely to engage in numeracy activities by themselves than younger children (e.g., because of homework from school), so it is important to gather this information as well.

In our opinion, scoring these response options as ‘1’ (the lowest non-0 option) makes the most sense because (i) we do not think any of these options are equivalent to the option “I did not engage in the activity” (they clearly indicate a somewhat more supporting HNE), but at the same time (ii) we do not want them to weigh excessively on the overall scoring (as our study is focused on present practices that are shared between parents and children). On p. 12, we now more clearly explain our rationale:

“For each activity, parents could choose among 8 response options. If they engaged in the activity with their child at home during the past month, they could indicate the frequency among 5 options: 1-3 times per month (scored 1), Once per week (scored 2), 2-4 times per week (scored 3), Almost daily (scored 4), Daily (scored 5). If they did not engage in the activity with the child within the past month, they had the choice between 3 response options. First, they could indicate that the child did practice the activity within the past month, but without parental involvement (as in 83). Second, they could indicate that they used to engage in the activity with the child in the past but no longer did at the time of testing. Adding these response options is important because children in the present study were older than in most previous studies on the HNE, which raises the possibility that they might engage in numeracy activity by themselves at home or that some activities in our questionnaires might no longer be relevant (19,62,83). It is thus critical to account for the reasons that might have led parents to not explicitly indicate that they engage in a given activity. In our main analyses, these two response options were each scored 1 because they still point to a somewhat supportive HNE, but we did not want them to weigh excessively on the overall scoring (as our study is focused on present practices that are shared between parents and children). However, we also present supplemental analyses in which these responses are kept separate to disentangle between present activities, past activities, and activities that children are doing alone. Third, the parents could also simply indicate that they did not engage in the activity or that the activity is not relevant. This last option was arguably not indicative of a supportive HNE and was therefore scored 0.”

That being said, we appreciate the reviewer’s point about being able to compare our results to other studies and we thank the reviewer for the suggestion. We have followed the advice and now present additional results in which present activities are dissociated from past activities and activities that children are doing alone. It is important, however, to consider that these two response options (i.e., past activities and activities that children are doing alone) remained presented to parents alongside the frequency scale of shared present activities. In other words, the mere presence of these options among the possible responses is likely to have influenced parents’ ratings and we cannot simply ignore this factor. Thus, we systematically added responses to past activities and responses to activities that children are doing alone as predictors of no interest in regression analyses investigating the effect of present activity shared with parents on math skills (see p. 18 for a description of the new analyses). We also added tables showing bivariate relations in supplementary materials, but these need to be interpreted with caution because the relation between present activities shared with parents and math skills is not controlled for the fact that other response options were presented.

On p. 22/23, we present the results of the new analyses in which present activities are dissociated from past activities and activities children were doing alone. As can be seen from the results, we still observed a significant relation between present activities and arithmetic fluency (there was only a trend for arithmetic calculation). This new paragraph reads:

“Second, as is standard in studies on the HNE, parents rated the frequency of present numeracy practices with children using a frequency scale. However, because the current study focuses on children who are older than in previous studies on the HNE, we also included two additional response options so that parents had the opportunity to indicate (i) that they used to engage in the activity in the past or (ii) that children engaged in the activity at present but without parental involvement. Although these response options weigh minimally on the calculation of the overall score (see Methods), we ran another set of analyses in which we dissociated present practices with parents from present practices without parents and past practices. S5 Table shows all bivariate relations when practices are dissociated, though these need to be interpreted with caution as all response options were presented concurrently (e.g., it is difficult to interpret a simple relation between skills and present practices with parent as two other response options were also available and not accounted for in such bivariate relations). When present practices with parent, present practices without parent and past practices were all included as separate predictors of multiple regression analyses, there remained a significant positive effect of present formal advanced numeracy practices on arithmetic fluency (SC = 0.484, 95% CI = [0.080, 0.888], p = 0.020) (S6 Table). The positive effect of present formal advanced numeracy practices on arithmetic calculation tended to be significant (SC = 0.392, 95% CI = [-0.020, 0.804], p = 0.061). These associations remained significant for arithmetic fluency (or near significant for arithmetic calculation) when we added in the models as control variables parental education and income (arithmetic fluency: SC = 0.469, 95% CI = [0.050, 0.889], t = 2.245, p = 0.029, η²p = 0.085; arithmetic calculation: SC = 0.401, 95% CI = [-0.025, 0.828], t = 1.887, p = 0.065, η²p =0.062;), child’s IQ (arithmetic fluency: SC = 0.481 95% CI = [0.081, 0.881], t = 2.411 p = 0.019, η²p = 0.096; arithmetic calculation: SC = 0.386, 95% CI = [0.002, 0.771], t = 2.012, p = 0.049, η²p = 0.069), parental math fluency (arithmetic fluency: SC = 0.510, 95% CI = [0.101, 0.919], t = 2.497, p = 0.016, η²p = 0.102; arithmetic calculation: SC = 0.393 95% CI = [-0.027, 0.812], t = 1.877, p = 0.066, η²p = 0.060), and number of hours spent with the child (arithmetic fluency: SC = 0.484, 95% CI = [0.076, 0.892], t = 2.375, p = 0.021, η²p = 0.093 arithmetic calculation: SC = 0.395, 95% CI = [-0.019, 0.810], t = 1.912, p = 0.061, η²p = 0.062). Therefore, present practices with parents uniquely contributed to the relation between formal advanced practices and arithmetic skills (particularly arithmetic fluency).”

On p. 26, we now also show that there remains a significant relation between parental advanced expectations and present practices (when past activities and activities that children are doing alone are excluded from the scoring). There we state:

“We then tested whether the relation between parental advanced expectations and formal practices hold when only present practices with parent are considered (i.e., when ratings associated with present practices without parent and past practices are excluded). S8 Table shows all bivariate relations when practices are dissociated. As can be seen on S9 Table, multiple regression analyses of present practices revealed that positive relations between parental advanced expectations and both formal basic practices (SC = 0.415, 95% CI = [0.121, 0.708], p = 0.006) and formal advanced practices (SC = 0.402, 95% CI = [0.108, 0.696], p = 0.008) remained positive. These associations remained significant when we added in the models as control variables parental education and income (formal basic practices: SC = 0.361, 95% CI = [0.071, 0.650], t = 2.491, p = 0.016, η²p = 0.095; formal advanced practices: SC = 0.402, 95% CI = [0.099, 0.704], t = 2.656, p = 0.010, η²p = 0.107), parental estimation of children’s numerical skills (formal basic practices: SC = 0.416, 95% CI = [0.120, 0.712], t = 2.810, p = 0.007, η²p =0.116; formal advanced practices: SC = 0.403, 95% CI = [0.106, 0.699], t = 2.713, p = 0.009, η²p = 0.109) and number of hours spent with the child (formal basic practices: SC = 0.427, 95% CI = [0.133, 0.721], t = 2.903, p = 0.005, η²p =0.123; formal advanced practices: SC = 0.409, 95% CI = [0.112, 0.705], t = 2.756, p = 0.008, η²p = 0.112).”

We thank the reviewer for their valuable feedback, and we hope that the new analyses will help other researchers use our results in future meta-analyses.
